# Depression, anxiety, and stress and fulfillment of contraceptive preferences among young people in Texas and California

**DOI:** 10.1371/journal.pone.0359942

**Published:** 2026-11-11

**Authors:** Hannah K. Hecht, Jennifer Yarger, Kristine Hopkins, Kristen Lagasse Burke, Irene Rossetto, Biftu Mengesha, Julia R. Steinberg, Luisa Alejandra Tello-Pérez, Madeline Molennor, Jennifer Kerns, Lucy Ogbu-Nwobodo, Cynthia C. Harper

**Affiliations:** 1 Department of Obstetrics, Gynecology and Reproductive Sciences, University of California, San Francisco, California, United States of America; 2 Philip R. Lee Institute for Health Policy Studies, University of California, San Francisco, California, United States of America; 3 Department of Epidemiology and Biostatistics, University of California, San Francisco, California, United States of America; 4 Population Research Center, The University of Texas at Austin, Austin, Texas, United States of America; 5 Department of Family Science, University of Maryland, College Park, Maryland, United States of America; 6 REACH Youth Study-Youth Advisory Board, San Francisco, California, United States of America; 7 Department of Psychiatry & Behavioral Sciences, University of California, San Francisco, California, United States of America; York University Faculty of Health, CANADA

## Abstract

**Background:**

Young people face both increased mental health concerns and growing barriers to accessing reproductive healthcare.

**Objective:**

This study examined the association of depressive, anxiety and stress symptoms with preferred contraceptive method use, a measure of healthcare access and reproductive autonomy.

**Methods:**

We used baseline data (April 2018-May 2023) from a cluster randomized trial at 29 community colleges in Texas and California. Participants were 2,065 sexually active, first-year community college students ages 18–25. We used multivariable mixed effects multinomial logistic regression for clustered data to examine the association of depressive (CESD-10) or anxiety and stress symptoms (DASS-21) and fulfillment of participants’ contraceptive preferences (unfulfilled, fulfilled or unknown), controlling for factors related to mental health and care access.

**Results:**

Overall, 51% of participants had an unfulfilled contraceptive preference, and 4% did not know what they preferred. Multivariable results showed elevated depressive symptoms (relative risk ratio [RRR]: 1.31, 95% confidence interval [CI]: 1.01, 1.71) or anxiety and stress symptoms (RRR: 1.31, 95% CI 1.03, 1.66) were associated with having an unfulfilled contraceptive preference versus a fulfilled preference. Additionally, elevated depressive symptoms were associated with having an unknown preference versus fulfilled preference (RRR: 2.98, 95% CI: 1.78, 4.99). Participants with mental distress were also significantly more likely to report cost or insurance barriers as reasons for having an unfulfilled preference (p < 0.05).

**Conclusions:**

Young people experiencing mental distress were less likely to realize their contraceptive preference. Future interventions should focus on providing mental health support and improving contraceptive access for young people, including addressing insurance barriers. A primary care model integrating both mental and reproductive healthcare will help to address important health needs of young people.

**Trial registration:**

**ClinicalTrials.gov Identifier:** NCT03519685

## Introduction

In the United States, mental distress symptoms are on the rise, particularly among adolescents, young adults, and those facing structural inequities [1,2]. Restrictive policies and barriers to reproductive health care also have increased, greatly impacting those from historically marginalized communities [3]. Most prior research on the mental health or reproductive health among college students has focused on those attending four-year universities [4,5] The few studies focused on community college students have shown that they are more likely to experience anxiety and depression [6] and have more limited access to mental and reproductive health resources and services, compared to their 4-year counterparts [7,8].

Previous studies examining the ties between mental health and contraception have centered on the associations between mental distress and current contraceptive method use or choices. Diagnosed depression and anxiety, as well as elevated depression, anxiety, and stress symptoms have been linked with contraceptive nonuse, less consistent use, discontinuation, and switching methods [9–11]. In some studies, mental distress was associated with choosing more effective methods [12,13], while in others it was associated with using less effective methods [11,14]. One study found that mental distress was linked with lower use of the pill, a method requiring mental energy for consistent use at a specified time [10]. Among college students in particular, having a history of depression has been associated with lower dual method use [15]. In this sizeable body of literature, however, no studies have examined the person-centered measure, fulfillment of contraceptive preferences.

Compared to current contraceptive method use, preferred contraceptive method use is a more person-centered metric of healthcare access as it better captures individual needs and desires [16,17]. Furthermore, preferred contraceptive method use has been conceptualized as a measure of reproductive autonomy [18–20]. Emerging research has shown that many people are not using their preferred contraceptive method [18]. For example, a recent nationally representative study found that among 15–44-year-olds assigned female at birth, one-quarter were not using their preferred method [16]. Prior studies have identified that socially and economically marginalized groups are more likely to have an unfulfilled contraceptive preference [21–23]. To date, research has not yet examined whether mental distress is related to fulfillment of contraceptive preferences, or the concordance between contraceptive preference and use.

Mental distress may present challenges for navigating health care and fulfilling contraceptive preferences. In Levesque’s theory of health care access, the ability to access care involves five dimensions: ability to perceive, ability to seek, ability to reach, ability to pay, and ability to engage [24]. Mental distress may come into play at various points along this continuum. For example, it may negatively impact the ability to seek or reach care, as it has been associated with delays in accessing contraceptive care and health care in general [25,26]. Additionally, mental distress may affect the ability to engage with care, which involves actively participating in care and decision-making. Mental distress may make it more challenging to develop clear contraceptive preferences, make decisions, and advocate for preferences [27].

In this study, we examined the association of symptoms of depression, anxiety and stress with unfulfilled contraceptive preferences among community college students, an understudied population with high mental and reproductive health needs. We hypothesized that mental distress would be associated with unfulfilled contraceptive preferences.

## Materials and methods

We used baseline survey data from the Reproductive Equity and Access in College Health (REACH Youth) study, a cluster randomized controlled trial testing a multi-level contraceptive intervention with health providers and students (ClinicalTrials.gov: NCT03519685). From April, 10 2018 to May 31, 2023, we enrolled 2,086 first-year students from 29 community colleges in California and Texas (please see S1 Table for the recruitment period for each college site). All community colleges in California and Texas were considered for inclusion in the study. Eligibility criteria required colleges to be accredited, serve students ages 18–25, not participate in other contraceptive interventions, and not have overlapping staff with other college sites. Colleges were primarily recruited through direct outreach to staff via email and phone, with additional support from organizations serving community colleges to facilitate connections. Of the 173 colleges assessed for eligibility, 31 were initially enrolled; one withdrew and another became ineligible after merging with another college site, resulting in 29 study sites.

Participants were eligible if they were 18–25 years old, assigned female sex at birth (gender inclusive), sexually active, not pregnant or desiring pregnancy in the next year, and were first-year students at an enrolled community college site. Of the 5,651 individuals assessed for eligibility, 2,877 were ineligible (2,130 were not sexually active, 755 were not first-year students, 237 were outside the age range, 88 were pregnant or desired pregnancy, 9 were assigned male at birth), and 688 chose not to enroll, with 2,086 enrolled in the study.

Participants were recruited through classroom announcements, emails from college staff, college websites, information tables, and flyers. Recruitment and enrollment occurred in-person until the start of the COVID-19 pandemic, when we adopted an exclusively remote approach. Over time, we resumed in-person recruitment and continued remote enrollment. Participants provided written consent through an electronic form and completed a baseline questionnaire with sociodemographic and economic characteristics, and reproductive and other health history, including mental health. After enrollment, participants received a $50 electronic gift card. We worked with our youth and community advisory boards to guide our approach to research, data interpretation, and dissemination efforts. This study was approved by the Institutional Review Boards (IRBs) at the University of California, San Francisco (#17–23183) and the University of Texas at Austin (#2019010078). Participating college sites either approved the study with their IRB (7 sites) or used the corresponding state university’s IRB (22 sites).

### Measures

The primary study outcome was whether participants’ contraceptive preferences were unfulfilled, fulfilled, or unknown. Participants were asked: “If you could use any birth control method that you wanted, what method would you use?” Response options included condoms, oral contraceptive pill, transdermal patch, vaginal ring, intrauterine device, sterilization, subdermal implant, injectable, fertility awareness, emergency contraception, withdrawal, other, and none, as well as “don’t know” and “I am using the method that I want.” We compared participants’ preferred contraceptive method with their current method. If participants were currently using more than one method, we used the one they reported was their main method. If participants reported more than one preferred method (n = 337), we used the preferred method that was the same as their current method (n = 141). Otherwise, we randomly assigned a preferred method from their selections (n = 196). We coded those who reported “I am using the method I want” or whose preferred method was the same as their current method as having a fulfilled preference, including no method if preferred. We coded participants who responded “don’t know” as having an unknown preference. We coded the remaining participants whose preferred method was different from their current method as having an unfulfilled preference. Among those with multiple preferred methods, none of which matched their current method, only n = 5 (out of n = 196) selected “don’t know” and could have been classified as having either an unfulfilled or unknown preference.

As a secondary outcome, we explored the reasons for having an unfulfilled contraceptive preference. Participants who were not using their preferred method were asked to select their reason(s) and could choose multiple response options, including an “other” write-in option. We categorized these reasons into system-level barriers, interpersonal- or individual-level barriers, and other barriers, following an approach used in Kavanaugh et al. (S2 Table) [17].

The independent measures of mental distress were elevated depressive symptoms and elevated anxiety and stress symptoms. To measure depressive symptoms, we used the 10-item Center for Epidemiological Studies Depression Scale (CESD-10) (e.g., *I felt depressed*, *My sleep was restless*) [28]. For anxiety and stress symptoms, we used seven items from the Depression Anxiety Stress Scales (DASS-21), including three items from the anxiety subscale (*heart action, difficulty breathing, and trembling*) and four from the stress subscale (*hard to wind down, overreacted, nervous energy, agitated/upset*) [29]. Participants rated how frequently they had experienced symptoms in the past two weeks on an adapted 4-point scale (never, rarely, sometimes, often). We summed the items to generate a score, imputing values for missing items by taking the average score of the non-missing items if participants answered at least seven CESD items and at least five DASS items [13]. We characterized participants as having elevated symptoms if their score was one standard deviation or greater above the mean [13]. We also tested using the recommended clinical cutoff of 10 or greater [30]. However, using this cutoff, most of the sample (83%) was characterized as having elevated symptoms, suggesting that this was not a sufficiently discriminating measure in our sample.

Our covariates were selected a priori based on sociodemographic and economic factors associated with mental health, access to health care, and contraceptive preferences [6,21,31]: Age (adolescents ages 18–19, young adults ages 20–25), has a child(ren) (yes/no), self-reported race and ethnicity (Hispanic/Latinx, White non-Hispanic, Black non-Hispanic, Asian/Pacific Islander non-Hispanic, American Indian/Multiracial/Other non-Hispanic), language spoken at home (English, Other), health insurance status (public, private, uninsured, don’t know), food insecurity as measured by how often their household worried about running out of food in the past month (sometimes/often true, never true) [32], and state of residence (California, Texas).

### Statistical analysis

From the full sample of enrolled participants (N = 2,086), we used listwise deletion to exclude participants missing data on contraceptive preference, elevated depression, anxiety, and stress symptoms, and covariates (approximately 1% of observations). The final analytic sample included 2,065 participants for analyses with depressive symptoms as the independent variable and 2,062 participants for analyses with anxiety and stress symptoms.

We described the distribution of participant characteristics overall and by elevated depression, anxiety, and stress symptoms. To compare characteristics between those with and without elevated symptoms, we used unadjusted mixed effects models with random effects for site. Then we estimated multivariable mixed effects multinomial logistic regression models with random effects for site to assess contraceptive preference (unfulfilled, fulfilled, unknown) by elevated depressive or anxiety and stress symptoms. The reference category in the multinomial models was fulfilled preferences, so the models compared those with unfulfilled vs. fulfilled preferences, and then those with unknown preference vs. fulfilled preferences. To adjust for potential confounding, all multivariable models included all covariates (age, race and ethnicity, language spoken at home, insurance status, food insecurity, parity, and state of residence). Using the multivariable models, we calculated the predicted probability of having an unfulfilled, fulfilled, or unknown contraceptive preference by elevated symptoms, adjusting for potential confounding.

Among participants with unfulfilled preferences, we explored their reasons for having an unfulfilled contraceptive preference by elevated mental distress symptoms. We presented the proportion of participants choosing each reason, by elevated depressive or elevated anxiety and stress symptoms. For statistical testing of differences in reasons given by elevated mental distress, we used unadjusted mixed effects models with random effects for site.

We conducted all analyses using Stata 18 and reported a significance level of *p* < 0.05.

## Results

The majority of participants were older adolescents 18–19 years old (82%) and identified as cisgender women (99%) and as heterosexual or straight (77%) (Table 1). Most participants (95%) did not have children, and 76% reported using contraception. Over half of the sample (58%) identified as Hispanic/Latinx, 21% as White, 10% as Asian or Pacific Islander, 6% as Black, and 6% as American Indian/Alaska Native, Multiracial, or other race, reflecting the community college student populations in California and Texas [33,34]. Half spoke a language other than English at home. Twenty-seven percent reported experiencing food insecurity, and 14% were uninsured.

**Table 1:** Participant characteristics, by elevated depressive, anxiety & stress symptoms (N = 2,065).

	Total %	Elevated depressive symptoms (n=315), 15.3 %	Elevated anxiety & stress symptoms (n=380), 18.4%
**Age**			
18-19	81.7	82.2	79.7
20-25 ^a^	18.3	17.8	20.3
**Gender identity**			
Female ^a^	99.1	98.4	98.4
Transgender/Non-binary/Oth	0.9	1.6	1.6
**Sexual orientation**			
Bisexual/Gay/Lesbian/Oth	23.0	35.9***	36.8***
Heterosexual/Straight ^a^	77.0	64.1	63.2
**Has child(ren)**	4.6	5.4	4.5
**Using contraception**			
None/withdrawal	23.7	28.9	25.5
Non-prescription/clinic-based method	35.8	31.1	34.0
Prescription/clinic-based method ^a^	40.5	40.0	40.5
**Race & ethnicity**			
Hispanic/Latinx	57.8	53.7	54.0*
White non-Hispanic ^a^	20.5	22.2	24.2
Black non-Hispanic	5.8	5.7	5.3
Asian/Pacific Islander non-Hispanic	10.7	11.1	10.3
Amer. Indian/Oth/Multi non-Hispanic	5.2	7.3	6.3
**Language other than English** **spoken at home**	50.0	44.4*	45.8
**Insurance status**			
No insurance	14.1	16.2	14.0
Public insurance	38.4	33.7	34.7*
Private insurance ^a^	36.3	39.1	41.8
Don’t know	11.2	11.1	9.5
**Food insecurity**	27.5	38.4***	40.3***
**State of residence**			
California ^a^	71.4	70.5	71.3
Texas	28.6	29.5	28.7

Note: Elevated symptoms: ≥ 1 standard deviation above the mean. * p < 0.05, ** p < 0.01, *** p < 0.001, Unadjusted mixed effects logistic regression models with random effects for site examined associations between participant characteristics and elevated mental distress symptoms.

^a^Reference category.

The 30-point depressive symptoms scale ranged from 0 to 29 and had a mean score of 15.3 (*SD* = 5.9); the 22-point anxiety and stress symptoms scale ranged from 0 to 21, with a mean score of 9.6 (*SD* = 5.3). Fifteen percent of our sample had elevated depressive symptoms, while 18% had elevated anxiety and stress symptoms. Unadjusted mixed-effects logistic regression analyses showed that elevated symptoms varied by participant sociodemographic and economic characteristics. Elevated depression, anxiety and stress symptoms were more likely among sexual minorities (bisexual, lesbian/gay, other) and those experiencing food insecurity (Table 1). Elevated depressive symptoms were less likely among those who spoke a language other than English, and anxiety and stress symptoms were less likely among those identifying as Hispanic/Latinx (compared to White non-Hispanic) and those with public insurance (compared to private). Overall, 51% of participants had an unfulfilled contraceptive preference, and 4% did not know what contraception they preferred.

We examined elevated mental distress symptoms as a predictor of unfulfilled contraceptive preference in multinominal models (Table 2). After adjusting for covariates, we found that elevated depressive symptoms were associated with a significantly increased likelihood of having an unfulfilled contraceptive preference (relative risk ratio [RRR]: 1.31, 95% confidence interval [CI]: 1.01, 1.71) and having an unknown preference (RRR: 2.98, 95% CI: 1.78, 4.99). In addition, elevated anxiety and stress symptoms were associated with increased risk of having an unfulfilled contraceptive preference (RRR: 1.31, 95% CI: 1.03, 1.66) but were not significantly associated with having an unknown preference. As a secondary finding, we observed that compared to White participants, those identifying as Hispanic/Latinx were more likely to have an unknown preference (Model 1: RRR 2.23, 95% CI: 1.03, 4.85; Model 2: RRR: 2.26, 95% CI: 1.04, 4.90). Finally, we calculated the predicted probability of having a fulfilled, unfulfilled, or unknown contraceptive preference from the estimates of these multivariable models (Figs 1 and 2).

**Table 2:** Multivariable mixed effects multinomial logistic regression models: Association of elevated mental depressive symptoms and anxiety and stress symptoms with fulfillment of contraceptive preference.

	Model 1 (N=2,065)		Model 2 (N=2,062)	
	Unfulfilled vs. fulfilled preference	Unknown vs. fulfilled preference	Unfulfilled vs. fulfilled preference	Unknown vs. fulfilled preference
	*RRR (95% CI)*	*RRR (95% CI)*	*RRR (95% CI)*	*RRR (95% CI)*
**Elevated depressive symptoms**	1.31 (1.01, 1.71)*	2.98 (1.78, 4.99)***	--	--
**Elevated anxiety & stress symptoms**	--	--	1.31 (1.03, 1.66)*	1.63 (0.95, 2.78)
**Age**				
18-19 years	1.13 (0.87, 1.46)	1.28 (0.68, 2.43)	1.15 (0.89, 1.48)	1.32 (0.70, 2.50)
20-25 years	Reference	Reference	Reference	Reference
**Race and ethnicity**				
Hispanic/Latinx	1.24 (0.94, 1.63)	2.23 (1.03, 4.85)*	1.24 (0.94, 1.64)	2.26 (1.04, 4.90)*
Black ^a^	1.32 (0.84, 2.07)	1.36 (0.39, 4.77)	1.32 (0.84, 2.07)	1.37 (0.39, 4.78)
Asian/PI ^a^	1.22 (0.84, 1.78)	2.23 (0.85, 5.83)	1.23 (0.85, 1.79)	2.24 (0.86, 5.85)
Amer. Indian/Oth/Multi ^a^	1.48 (0.95, 2.31)	1.59 (0.46, 5.47)	1.50 (0.96, 2.34)	1.70 (0.50, 5.80)
White	Reference	Reference	Reference	Reference
**Language other than English at home**	1.29 (1.03, 1.61)*	1.09 (0.65, 1.83)	1.29 (1.03, 1.61)*	1.04 (0.62, 1.74)
**Insurance status**				
No insurance	1.35 (0.98, 1.84)	1.29 (0.63, 2.64)	1.35 (0.99, 1.85)	1.33 (0.65, 2.72)
Public insurance	1.10 (0.87, 1.38)	1.16 (0.65, 2.04)	1.10 (0.88, 1.39)	1.13 (0.64, 1.99)
Don’t know	1.27 (0.92, 1.75)	1.81 (0.91, 3.61)	1.27 (0.93, 1.76)	1.85 (0.93, 3.68)
Private	Reference	Reference	Reference	Reference
**Food insecure**	1.15 (0.93, 1.42)	0.92 (0.54, 1.54)	1.14 (0.92, 1.41)	0.96 (0.57, 1.62)
**Has child(ren)**	0.56 (0.35, 0.90)*	0.31 (0.07, 1.41)	0.57 (0.35, 0.92)*	0.33 (0.07, 1.48)
**State of residence**				
Texas	1.81 (1.30, 2.52)***	2.20 (1.10, 4.43)*	1.80 (1.29, 2.50)***	2.21 (1.12, 4.37)*
California	Reference	Reference	Reference	Reference

RRR = relative risk ratio estimated from mixed effects multinomial logistic regression models with random site effects; CI = confidence interval; * p < 0.05, **p < .010 ***p < 0.001

^a^Non-Hispanic

**Fig 1:**
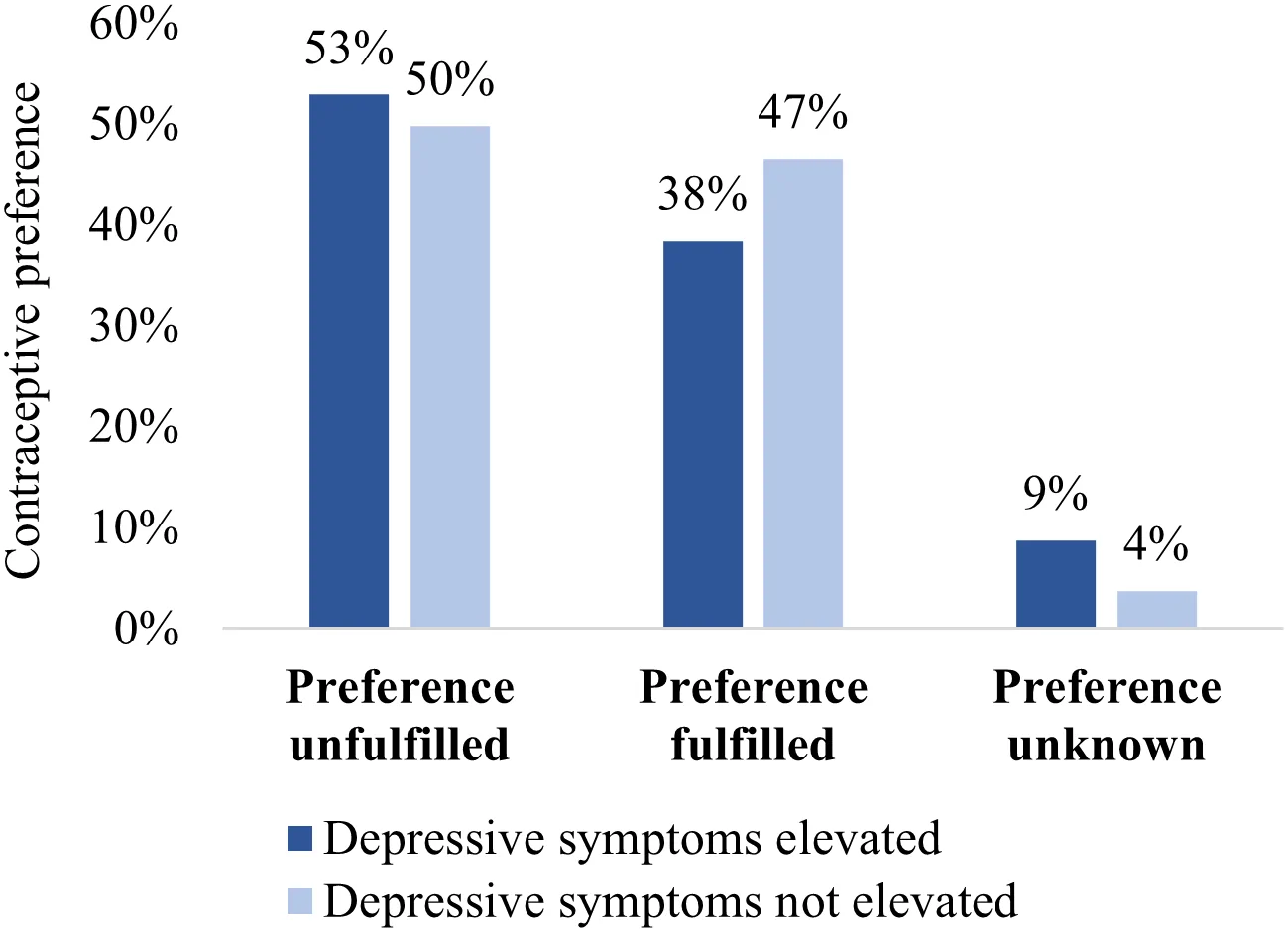
Predicted probability of contraceptive preference, by elevated depressive symptoms. Note: Elevated symptoms: ≥ 1 standard deviation above the mean. Results are predicted probabilities from Model 1 (N = 2,065) accounting for covariates (age, race and ethnicity, language spoken at home, insurance status, food insecurity, parity, and state of residence).

**Fig 2:**
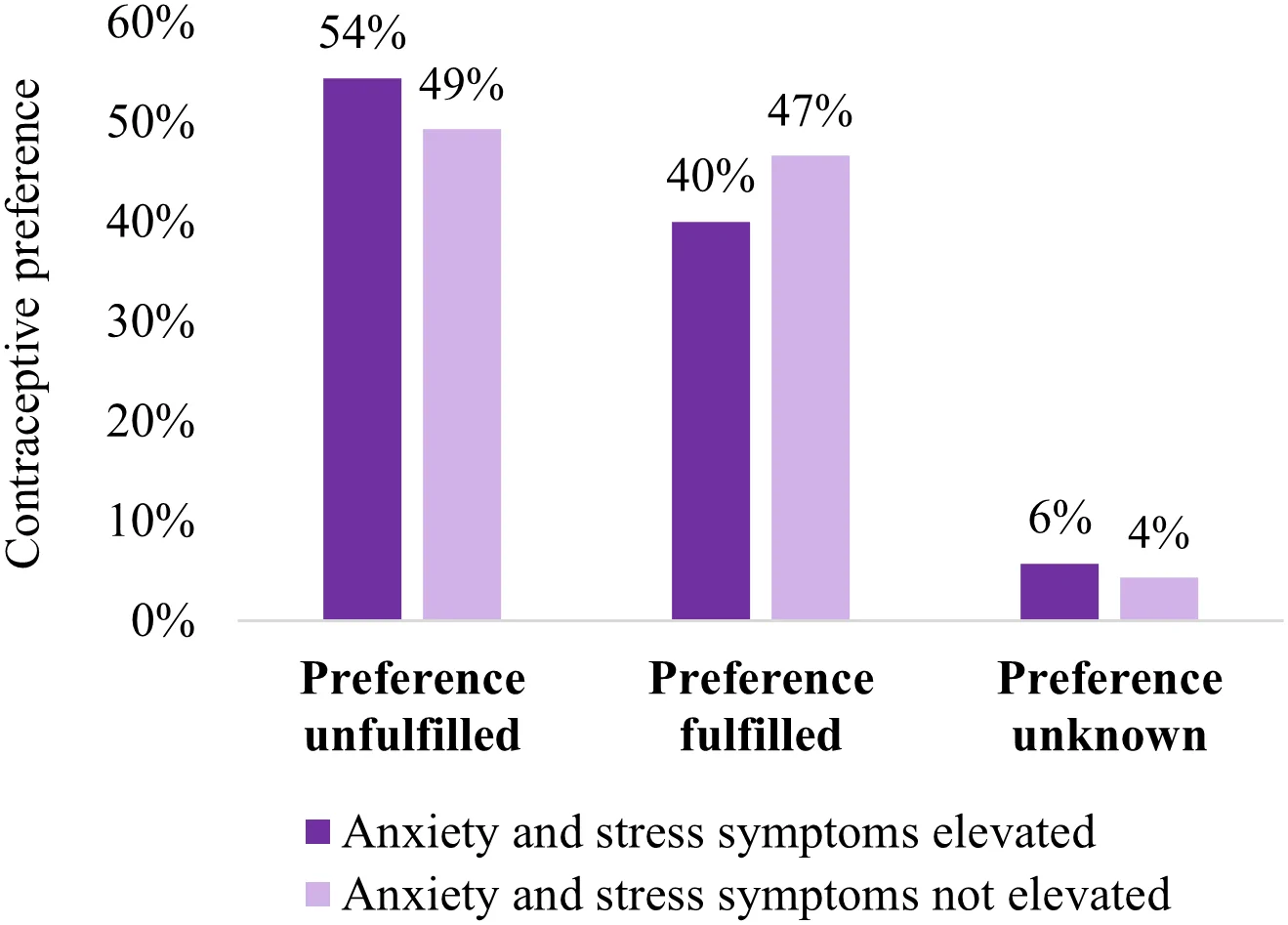
Predicted probability of contraceptive preference, by elevated anxiety and stress symptoms. Note: Elevated symptoms: ≥ 1 standard deviation above the mean. Results are predicted probabilities from Model 2 (N = 2,062) accounting for covariates (age, race and ethnicity, language spoken at home, insurance status, food insecurity, parity, and state of residence).

We conducted two sensitivity analyses, one assigning all n = 5 participants with multiple preferred methods including “don’t know” to having an unfulfilled preference and another assigning them to an unknown preference; the results were consistent with those from the randomization approach.

Participants reported reasons for having an unfulfilled contraceptive preference at the system and individual levels, which we present by elevated mental distress symptoms (Table 3). Overall, the most common system level reasons were clinic or method access (ranging from 50% to 54%) and cost or insurance barriers (23%−34%). The most common individual or interpersonal level was parent or partner barriers (33%−37%). In unadjusted mixed effects models, those with elevated symptoms were significantly more likely to report cost and insurance barriers, compared to those without elevated symptoms (Depressive symptoms: 34% vs. 23%; p < 0.01; Anxiety and stress symptoms: 31% vs. 23%; p < 0.05). Additionally, those with elevated anxiety and stress symptoms were significantly more likely to list side effects as a reason for not using their preferred method, compared to those without elevated symptoms (15% vs. 9%; p < 0.01).

**Table 3:** Reasons for having an unfulfilled preference, by elevated symptoms of depression or anxiety and stress.

	Elevated depressive symptoms, %		Elevated anxiety & stress symptoms, %	
	Yes (n=166)	No (n=841)	Yes (n=199)	No (n=805)
**System-level barriers**				
Clinic or method access barriers	54.2	49.8	52.8	50.1
Cost or insurance barriers	34.3**	23.3	31.2*	23.4
Provider barriers	5.4	7.3	8.0	6.7
**Individual or interpersonal barriers**				
Parent or partner barriers	33.7	37.0	33.2	37.4
Side effects ^a^	12.0	9.4	15.1**	8.6
No perceived need	11.5	11.2	9.6	11.7
**Other**	7.8	6.4	8.5	6.2

* p < 0.05, ** p < 0.01, This table presents the percentages of participants who choose each reason by elevated mental distress. We used unadjusted mixed effects logistic regression with random effects for site to examine the associations between each reason and elevated mental distress. See S2 Table for examples of each reason.

^a^Side effects captures fear of future side effects (e.g., “I’m scared to gain weight”) as well as past experiences with side effects (e.g., “It affected my weight”).

## Discussion

In this study of community college students with overall high levels of unfulfilled contraceptive preference, we found that adolescents and young adults with elevated depressive or anxiety and stress symptoms were even more likely to have an unfulfilled contraceptive preference compared to a fulfilled preference. To our knowledge, this study is the first to examine the association of mental distress with preferred contraceptive method use, a person-centered measure of health care access and reproductive autonomy [16,20]. Prior studies of mental distress have focused on its association with contraceptive use or choices rather than fulfillment of contraceptive preferences [9,13]. Meanwhile, previous studies on predictors of preferred method use have examined various sociodemographic and care quality factors, but not mental distress [16,18,22].

Some participants were unsure of their contraceptive preference, which is consistent with prior research [16]. By identifying those with an unknown preference as a distinct group, our study expands on prior research, which has largely focused on a binary indicator of unfulfilled preference. Analyzing those with unknown preferences separately showed that those with elevated depressive symptoms had greater than twice the likelihood of having an unknown preference. Addressing this group’s needs is important, as identifying one’s preference is a key step toward fulfilling it.

Our main finding that elevated mental distress symptoms were associated with not using a preferred contraceptive method may reflect how mental distress comes into play in Levesque’s health care access model [24]. Although we did not directly measure this dimension of access, we theorize that mental distress symptoms may affect the ability to seek care. In another study using longitudinal data from this sample, we found that mental distress was associated with young people delaying contraceptive care [25]. Other studies have linked mental distress with delays in accessing healthcare overall [26,35]. Young people with mental distress may be less likely to use their preferred method due to delaying care needed to access that method. Second, certain symptoms of mental distress, such as low energy and motivation, may make it more challenging not only to seek care but also to engage with healthcare [27]. Those with depressive symptoms may be more likely to have an unknown preference due to challenges accessing healthcare to learn about their options, perhaps as a result of reduced self-efficacy [36]. They also may experience difficulty with making decisions about preferences without adequate support, a common cognitive symptom of depression [37]. Depressive symptoms could also be associated with unknown preferences due to a reluctance to seek care for sensitive health topics. Our youth advisory board discussed how both mental health and reproductive health issues are stigmatized, and it may be especially challenging for young people to seek care on both fronts. Finally, individuals who have overall limited access to healthcare might have difficulty finding treatment for depression or exposure to comprehensive counseling and education on reproductive health (i.e., without interacting with a provider, it could be challenging to treat depression and develop contraceptive preferences). Outreach to youth populations for mental health and reproductive health needs is warranted, as are continued efforts to equip primary care providers to address youth mental and reproductive health.

The reasons participants reported for not using their preferred method provide insights into the relationship between mental distress and preferred method use. Overall, many participants cited clinic or method access barriers, along with cost and insurance barriers, which is consistent with prior research [31]. Affordability of contraception is a significant barrier for the community college student population [38]. Those experiencing mental distress may have more difficulty accessing or using insurance and programs that help cover the cost of contraceptive services, which may help explain why they were more likely to report cost or insurance barriers. We also found that concerns about side effects were significantly more common among those with elevated anxiety and stress symptoms, an important finding for clinical care and counseling. There is mixed empirical evidence about whether mood side effects of hormonal contraception may be more likely among those with pre-existing mental disorders, though these studies have most commonly examined depression [39].

This study has limitations. We examined symptoms of mood disorders and are not able to capture whether other types of mental illness relate to unfulfilled preferences. We also measured self-reported symptoms and did not have a measure of depression or anxiety diagnosis. For our measures of mental distress, we used one standard deviation or greater above the mean as our cutoff for elevated symptoms, which may limit the generalizability of our findings and excludes those below this threshold who still have meaningful symptoms. However, with the established clinical cutoff for depression [30], the vast majority of our sample was characterized as having elevated symptoms. We chose to use one standard deviation or greater as the cutoff as it has been used in prior studies [12,13] and was more discriminating than the established clinical cutoff, providing insights into the contraceptive experiences of those with the most severe mental distress symptoms. Overall, the large proportion of clinically significant symptoms in our sample highlights the importance of further studies of mental distress in community college populations, which may be at elevated risk compared to their 4-year counterparts [6,8]. Although our models adjusted for several sociodemographic factors likely to be strong confounders, there were certain variables that we were not able to account for, such as past experiences with side effects or providers. In addition, in our measure of reasons for an unfulfilled preferences, “concerns about side effects” emerged as a category from the write-in option. A greater number of participants might have reported concerns about side effects had it been included as a separate response option. Given that this analysis used only baseline data, we were not able to measure how participants’ contraceptive preferences may have changed over time. Future studies are needed to examine how mental distress impacts fulfillment of contraceptive preferences longitudinally.

This study also has notable strengths. We used a robust measure of unfulfilled contraceptive preference that does not assume participants know their preferred method, as it includes “don’t know” as a response category. Our youth advisory board members have shared that it may be challenging to identify a preferred method, especially without comprehensive education. Our study focuses on an understudied population of community college students living in states with different reproductive healthcare policies and levels of affordable care. The association between mental distress and unfulfilled or unknown preferences stood across state contexts. However, in Texas, a state with greater reproductive health care restrictions than California, participants overall had lower preferred method use and greater unknown contraceptive preferences [21]. We identified greater unknown preferences among Hispanic/Latinx participants compared to White. This finding may reflect barriers to information about contraception necessary for identifying a preference, such as reduced access to health care services or adverse experiences with contraceptive counseling [40,41]. Future qualitative studies may be helpful for understanding the mechanisms underlying individuals’ unknown preferences and how to facilitate the fulfillment of their preferences. Our findings have important implications for clinical practice and healthcare systems. They highlight the importance of mitigating system-level and individual or interpersonal-level barriers to preferred method access among young people, especially those experiencing mental distress. Providers should be aware that young people with mental distress may experience greater concerns about side effects and more financial barriers to accessing their preferred method. Providers should also be mindful that those with mental distress may be less likely to know what their preferred method is, thus requiring support to develop a preference. Our findings therefore suggest that integrating mental health screenings into reproductive health care services could help providers tailor care to their patients’ needs.

Reproductive and mental health services tend to be siloed. However, reproductive healthcare providers can be main healthcare access points for many [42]. One study found that while most women preferred to go to primary care provider or mental health specialist, a meaningful proportion would prefer to get primary and mental healthcare from their reproductive health provider, particularly among women in minoritized communities [43]. Our youth advisory board members have echoed this preference for integrated care, emphasizing it could be less burdensome for those experiencing mental distress. Community college students might benefit from the convenience of the co-location of reproductive and mental health services, given that many students tend to have limited time (e.g., they are balancing school with other responsibilities, such as work) [44]. Yet, there have been few efforts to integrate sexual and reproductive care into mental healthcare for young people [45,46]. Moreover, with recent changes in federal policy and resource allocations, mental and reproductive healthcare services face significant setbacks, which are anticipated to have adverse impacts on mental and reproductive health outcomes. To promote access to mental healthcare as well as information about contraceptive options and preferred methods, future policies, clinical interventions, and healthcare settings should aim to expand integrated mental health and reproductive services for young people, particularly for those facing barriers to accessing care.

## Supporting information

S1 Table Dates of recruitment for each study site. Note: The COVID-19 pandemic occurred about two years into the study, which affected the pace of enrollment.
(DOCX)

S2 Table Reasons for having an unfulfilled contraceptive preference. Note: *Coded from write-in responses to the survey response option, “Other (please specify):”; Participants could provide multiple reasons for having an unfulfilled preference (N = 1,478 responses from 1,007 individuals).
(DOCX)
