# Supplementary material for: Depression, anxiety, and stress and fulfillment of contraceptive preferences among young people in Texas and California
Source: PLoS One. 2026 Nov 11;21(11):e0359942. doi: 10.1371/journal.pone.0359942 (PMC13754137; doi:10.1371/journal.pone.0359942)
Supplement: Supplementary file 1 [file pone.0359942.s001.docx]

**S1 Table. Dates of recruitment for each study site.**

| **Site Number** | **State** | **Recruitment start date**  **(DD/MM/YYYY)** | **Recruitment**  **end date**  **(DD/MM/YYYY)** |
| --- | --- | --- | --- |
| 1 | California | 09/04/2018 | 28/02/2019 |
| 2 | California | 23/04/2018 | 17/05/2019 |
| 3 | California | 07/05/2018 | 13/09/2019 |
| 4 | California | 10/09/2018 | 22/02/2019 |
| 5 | California | 17/09/2018 | 28/09/2018 |
| 6 | California | 24/09/2018 | 11/04/2019 |
| 7 | California | 24/09/2018 | 08/11/2018 |
| 8 | California | 26/09/2018 | 17/05/2019 |
| 9 | California | 01/10/2018 | 01/02/2019 |
| 10 | California | 22/10/2018 | 14/02/2019 |
| 11 | California | 19/02/2019 | 12/04/2019 |
| 12 | California | 25/03/2019 | 08/11/2019 |
| 13 | California | 03/09/2019 | 11/10/2019 |
| 14 | California | 23/09/2019 | 18/10/2019 |
| 15 | Texas | 22/10/2019 | 11/12/2019 |
| 16 | Texas | 28/10/2019 | 11/12/2019 |
| 17 | California | 11/11/2019 | 06/03/2020 |
| 18 | California | 13/01/2020 | 28/05/2021 |
| 19 | Texas | 27/01/2020 | 15/12/2020 |
| 20 | California | 03/02/2020 | 12/01/2023 |
| 21 | Texas | 02/03/2020 | 18/11/2020 |
| 22 | California | 01/06/2020 | 19/04/2023 |
| 23 | Texas | 15/10/2020 | 31/05/2023 |
| 24 | California | 19/01/2021 | 07/03/2023 |
| 25 | Texas | 25/02/2021 | 31/05/2023 |
| 26 | Texas | 05/05/2021 | 31/05/2023 |
| 27 | Texas | 27/09/2021 | 31/05/2023 |
| 28 | Texas | 03/03/2022 | 31/05/2023 |
| 29 | California | 05/04/2022 | 22/03/2023 |

Note: The COVID-19 pandemic occurred about two years into the study, which affected the pace of enrollment.
