# Supplementary material for: Depression, anxiety, and stress and fulfillment of contraceptive preferences among young people in Texas and California
Source: PLoS One. 2026 Nov 11;21(11):e0359942. doi: 10.1371/journal.pone.0359942 (PMC13754137; doi:10.1371/journal.pone.0359942)
Supplement: Supplementary file 2 [file pone.0359942.s002.docx]

**S2 Table. Reasons for having an unfulfilled contraceptive preference.**

| *Domains and categories* | N |
| --- | --- |
| **System-level barriers** | **832** |
| Clinic/method access | 509 |
| It’s too much hassle to get it  I don’t know where I can get it  I have an appointment scheduled to get it soon  I don’t have enough information*  *Examples: I don’t know enough about it; Never heard of it until now* | 304  213  64  16 |
| Cost/insurance barriers | 253 |
| I can’t afford it  My insurance doesn’t cover it | 216  98 |
| Provider barriers | 70 |
| My doctor advised against it  My doctor/clinic doesn’t offer it  Sterilization barriers*  *Examples: They will not tie my tubes because of my age;  Most doctors will not perform unless already had a child* | 46  24  4 |
| **Individual-level or interpersonal-level barriers** | **579** |
| Parent/partner barriers | 367 |
| My parents don’t know I’m sexually active  My partner doesn’t want me to use it  Other parent or family barriers*  *Examples: My parents don’t want me to use it; Discouragement from family* | 331  46  8 |
| No perceived need | 113 |
| I’m not sexually active  Don’t need/don’t want/already tried*  *Examples: I don’t need to use it, I don’t want it,*  *I tried it and it did not work for me* | 95  20 |
| Side effects* | 99 |
| *Examples: Scared of side effects; It’s painful; Birth control messed up my period* |  |
| **Other barriers*** | **67** |
| *Examples: Not sure if I want it yet; I like the one I’m using; Religion; I wouldn’t be good at tracking everything* |  |

Note: *Coded from write-in responses to the survey response option, “Other (please specify):”; Participants could provide multiple reasons for having an unfulfilled preference (N=1,478 responses from 1,007 individuals).
